# Seroprevalence of Seven Reproductive Diseases in Beef and Dairy Cows from Three Provinces in Indonesia

**DOI:** 10.1155/2021/6492289

**Published:** 2021-12-02

**Authors:** Didik Tulus Subekti, Mira Fatmawati, Arie Khoiriyah, Arum Pramesthi, Sulinawati Fong, Muhammad Ibrahim Desem, Zul Azmi, Eni Kusumaningtyas, Dwi Endrawati, Eko Setyo Purwanto

**Affiliations:** ^1^Indonesian Research Center for Veterinary Science, Jalan R.E. Martadinata No. 30, Bogor 16114, West Java, Indonesia; ^2^Faculty of Veterinary Medicine, Brawijaya University, Jalan Puncak Dieng, Dau Sub-District, Malang 65151, East Java, Indonesia; ^3^Veterinary Disease Investigation Center, Jalan Untung Suropati No. 2, Labuhan Ratu, Bandar Lampung 35142, Lampung, Indonesia; ^4^Goats Research Station, P.O. Box 1 Galang, Sei Putih 20585, North Sumatra, Indonesia

## Abstract

Bovine reproductive diseases are endemic in Indonesia, but comprehensive information about their infectious causes is not available. Therefore, our aim for this study was to detect several infectious agents that cause reproductive diseases in Indonesian beef and dairy cows. A total of 152 cow serum samples collected by Faculty of Veterinary Medicine of Brawijaya University and Veterinary Disease Investigation Centre as a part of the mandatory and regularly surveillance system from three provinces during 2019–2020 were used. The samples were then sent to Indonesian Research Centre for Veterinary Science (IRCVS) for further detection of seven reproductive diseases by enzyme-linked immunosorbent assay (ELISA). Seven reproductive diseases to be tested in parallel are neosporosis, chlamydiosis, brucellosis, Q fever, bovine viral diarrhea (BVD), infectious bovine rhinotracheitis (IBR), and BHV-4 infection. The dominant reproductive diseases in Indonesian cows were BVD (45.69%), chlamydiosis (31.58%), IBR (20.53%), neosporosis (11.84%), and BHV-4 infection (10.53%). The seroprevalence of IBR, BHV-4 infection, neosporosis, and brucellosis varied significantly (*P* < 0.05) between dairy and beef cattle. The most dominant reproductive diseases in aborted cows were chlamydiosis (45%), BVD (41%), and neosporosis (10%). The conclusion drawn from this study is that the dominant reproductive diseases in Indonesian cows are BVD, chlamydiosis, IBR, neosporosis, and BHV-4 infection. Chlamydiosis, BVD, and neosporosis are common among aborted cow. Chlamydiosis, neosporosis, and BHV-4 infection should be included in the national priority list in Indonesia. Control and preventive measures should be focused on high-risk areas and animals like stray cat and dog.

## 1. Introduction

Loss of pregnancy in either beef or dairy cows causes a lot of economic loss. In general, the causes of loss of pregnancy in cows can be caused by infectious or noninfectious factors. Common noninfectious causes are poisoning, hormonal imbalances, trauma, and poor nutrition [1, 2]. The infectious causes of abortions are very diverse, either viral, bacterial, or protozoal. The exact proportion of abortion cases in cows due to infectious agents is unknown. However, in 90% of the cases, when an etiological diagnosis is reached, it is caused by an infectious agent [2, 3].

Abortions in dairy cows in Indonesia are reported to be 2.96% in dairy farming companies in West Java province [4] and 41.07% in National Centres for Livestock Breeding in Central Java province [5]. These percentages are greater than the percentage of abortion cases in dairy cows in Ethiopia which ranges from 2.20 to 14.60% [6]. Abortion cases in dairy cows in Australia and New Zealand are only 7% and 6%, respectively [7]. However, the cases of abortion in Balinese and Madurese cattle in Indonesia were reported to be 0.77%, respectively [8].

Comprehensive information of reproductive diseases like abortion are endemic in Indonesia, but their infectious causes are not known. Generally, the prevalence data for bovine reproductive diseases in Indonesia are dominated by brucellosis prevalence studies. However, brucellosis alone is not always responsible for reproductive diseases. Some other infectious agents that can cause loss of pregnancy in cows are *Neospora caninum* [9–11], *Chlamydophila abortus*, *Chlamydophila pecorum* [11, 12], *Coxiella burnetii* [11, 12], bovine viral diarrhea (BVD) [11–13], bovine herpesvirus type 1 (BHV-1) [7, 13], and bovine herpesvirus type 4 (BHV-4) [11, 14]. Therefore, studies to determine the infectious causes of reproductive diseases are very important in Indonesia. Hence, the objective of this study was to detect several infectious agents that cause reproductive diseases in Indonesian beef and dairy cows.

## 2. Materials and Methods

### 2.1. Study Design and Samples History

A total of 152 cow serum samples collected by Faculty of Veterinary Medicine of Brawijaya University (FVMBU) and Veterinary Disease Investigation Centre (VDIC) as a part of the mandatory and regularly surveillance system from three provinces during 2019–2020 were used. All samples were previously tested by FVMUB and VDIC with negative results for brucellosis using RBT (rose bengal test) and CFT (complement fixation test) methods. The samples were then sent to Indonesian Research Centre for Veterinary Science (IRCVS) for further detection of seven reproductive diseases by enzyme-linked immunosorbent assay (ELISA). Seven reproductive diseases to be tested in parallel are neosporosis, chlamydiosis, brucellosis, Q fever, bovine viral diarrhea (BVD), infectious bovine rhinotracheitis (IBR), and BHV-4 infection.

One hundred and fifty-two samples of bovine serum consisted of 88 samples from dairy cows and 64 samples from beef cattle. All samples from dairy cow are female Friesian Holstein (FH) originating from East Java (72 samples), West Java (48 samples), and Lampung (32 samples). The samples of beef cattle from East Java were females, Peranakan Ongole (PO), and all samples from Lampung were female PO except for only two females of Simental and Brangus. Among the 88 samples of dairy cows, there were 16 samples with a history of abortion, while from the 64 samples of beef cattle, there were 8 samples experiencing abortions. Animal ethics committee approval and explicit consent were not required from the farmers as the study was part of mandatory and regularly surveillance and serum collection from the Veterinary Diseases Investigation Centre.

### 2.2. Rose Bengal and Complement Fixation Test

The RBT and CFT were only performed for the diagnosis of brucellosis. The RBT was carried out using the reagent from Pusat Veteriner Farma, Indonesia, according to the manufacturer's user manual. The CFT was carried out based on the standard testing protocol established by OIE [15].

### 2.3. Enzyme-Linked Immunosorbent Assay

ELISA was carried out for the diagnosis of seven reproductive diseases such as neosporosis (*Neospora caninum*), brucellosis (*Brucella abortus*), chlamydiosis (*Chlamydophila abortus*), Q fever (*Coxiella burnetii*), bovine viral diarrhea (BVD), infectious bovine rhinotracheitis (IBR) caused by the bovine alphaherpesvirus 1 (BHV-1), and the infection caused by the bovine gammaherpesvirus 4 (BHV-4). Commercial ELISA kits were used for serological tests against *Neospora caninum* (IDEXX Neospora Ab Test, Switzerland), *Brucella abortus* (IDEXX Brucellosis Serum Ab Test, France), *Chlamydophila abortus* (IDEXX Chlamydiosis Total Ab Test, Switzerland), *Coxiella burnetii* (IDEXX Q Fever Ab Test, Switzerland), BVDV (IDEXX BVDV Total Ab Test, Switzerland), BHV-1 (IDEXX IBR gB X3 Ab Test, Switzerland), and BHV-4 (ID Screen® BHV-4 Indirect, France). The ELISA tests were carried out according to the manufacturer's user manual.

### 2.4. Statistical Analysis

Data obtained were presented descriptively and analyzed statistically using Epitools and MedCalc version 19. The variations in the prevalence between dairy cows and beef cattle and among different regions were evaluated using the chi-square test. The level of significance was set at *P* < 0.05.

## 3. Results

### 3.1. Regional Distribution of Reproductive Diseases

**Figure 1:**
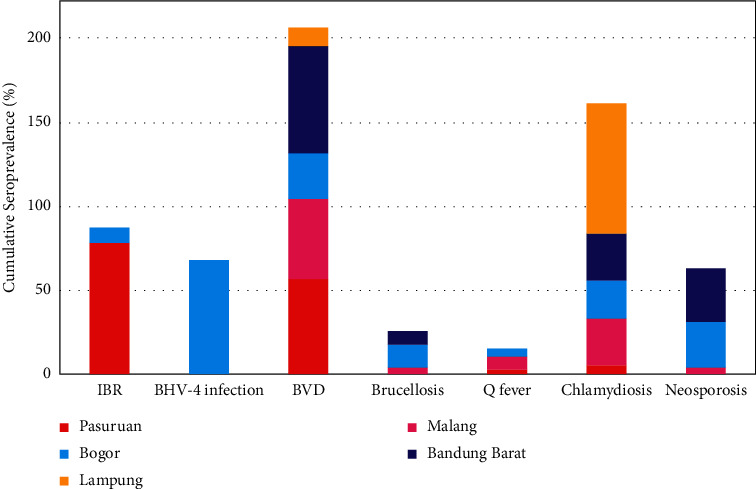
Seroprevalence of seven reproductive diseases in female cows from five cities in Indonesia.

The dominant reproductive diseases in East Java province were IBR, BVD, and chlamydiosis with a prevalence of more than 10% (Table 1 and Figure 1). The seroprevalence of IBR (78.38%) and BVD (56.76%) was the highest in Pasuruan, East Java. In Batu, East Java, the dominant reproductive diseases were BVD (48%) and chlamydiosis (28%).

Five reproductive diseases were detected with a seroprevalence greater than 10% in West Java province: BHV-4, BVD, neosporosis, chlamydiosis, and brucellosis. All five were found in Bogor, West Java. In descending order, the seroprevalence of reproductive diseases in Bogor was 68.18% for BHV-4 infection, 27.27% for both neosporosis and BVD, 23.81% for chlamydiosis, and 13.64% for brucellosis. Meanwhile, in West Bandung, the highest seroprevalence was 64% for BVD, followed by 32% (neosporosis), 28% (chlamydiosis), and 8% (brucellosis).

The highest seroprevalences in Lampung province were for chlamydiosis and BVD at 77.78% and 11.11%, respectively. The seroprevalence of chlamydiosis in Lampung Province (77.78%) was significantly (*P* < 0.01) higher compared to other locations (5.26%–28%). The BVD seroprevalence in Lampung Province (11.11%) was significantly (*P* < 0.05) lower from the BVD seroprevalence in Pasuruan (56.76%) and West Bandung (64%) only.

The reproductive diseases most commonly detected in five cities were BVD and chlamydiosis (Figure 1), followed by neosporosis (detected in four cities) and brucellosis and Q fever (detected in three cities), respectively. As for IBR and BHV-4 infection, although their seroprevalence was quite high, they were limited to one location only, so they were considered as a specific problem for that region. IBR was only dominant in Pasuruan, and BHV-4 infection was only dominant in Bogor.

### 3.2. The Seroprevalence of Reproductive Diseases in Dairy and Beef Cattle

**Figure 2:**
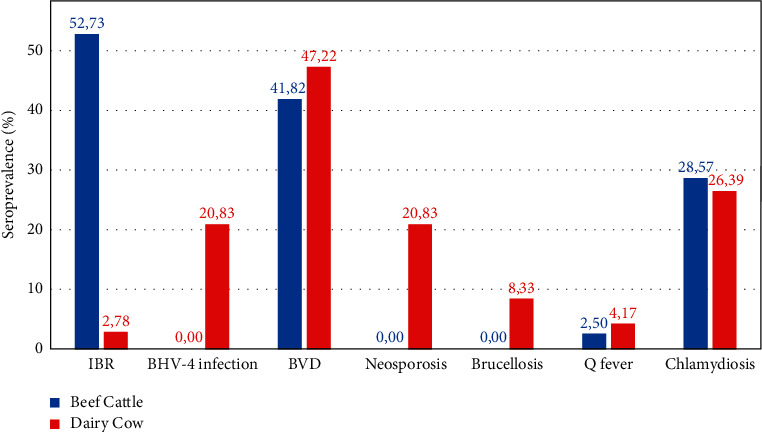
Seroprevalence of seven reproduction diseases in female beef cattle (blue bar) and dairy cow (red bar).

Beef cattle showed seropositivity to four reproductive diseases, namely, IBR, BVD, chlamydiosis, and Q fever (Figure 2). The prevalence of IBR, BVD, chlamydiosis, and Q fever in beef cattle was 52.73%, 41.82%, 28.57%, and 2.50%, respectively. On the other hand, seven reproductive diseases were detected in dairy cows with high seroprevalence (greater than 10%) in four diseases: BVD (47.22%), chlamydiosis (26.39%), neosporosis (20.83%), and BHV-4 infection (20.83%), as shown in Figure 2. A significant difference in seroprevalence (*P* < 0.05) was found between beef cattle and dairy cow in four reproductive diseases: IBR, BHV-4 infection, neosporosis, and brucellosis.

### 3.3. The Proportion of Reproductive Diseases in Seropositive Cows

**Figure 3:**
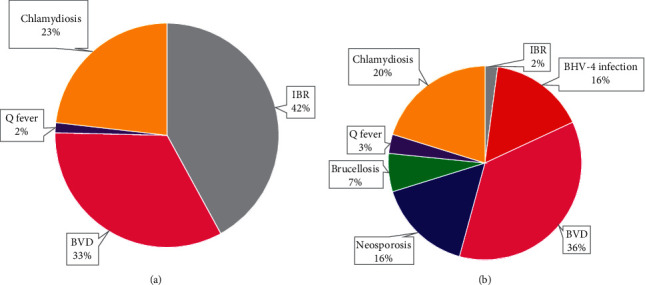
Proportions of each reproductive diseases among seropositive female beef cattle (a) and dairy cow (b).

Among the 64 beef cattle, 57 cattle were declared as seropositive for one or more reproductive diseases. In total, there were 78 cases of four reproductive diseases that were detected in 57 seropositive cows. The proportion of four reproductive diseases from the 78 seropositive cases in beef cattle was dominated by IBR (42%), BVD (33%), and chlamydiosis (23%), as shown in Figure 3(a).

In contrast, 63 of 88 dairy cows were stated seropositive for one or more reproductive diseases. In total, there were 114 cases of seven reproductive diseases that were detected in 63 seropositive cows. The proportion of seven reproductive diseases from 114 seropositive cases in dairy cows was dominated by BVD (36%), chlamydiosis (20%), neosporosis (16%), and BHV-4 infection (16%), as shown in Figure 3(b).

**Figure 4:**
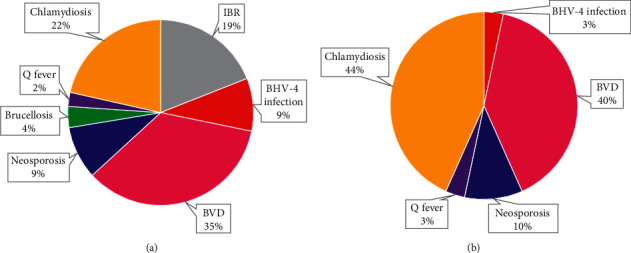
Proportions of each reproductive disease among seropositive cows (a) without the history of abortion and (b) with the history of abortion.

The results of this study found that 192 cases of seropositive reproductive diseases were distributed among 96 healthy cows with no history of abortion, but had been declared seropositive to one or more reproductive diseases. Proportionally, the five most dominant reproductive diseases in healthy cows were BVD (35%), chlamydiosis (22%), IBR (19%), BHV-4 infection (9%), and neosporosis (9%), as shown in Figure 4(a). The three most dominant reproductive diseases in cows with the history of abortion were chlamydiosis (44%), BVD (40%), and neosporosis (10%), as shown in Figure 4(b).

Table 2 provides the seroprevalence of reproductive diseases in cows with and without the history of abortion. The seroprevalence for BVD, chlamydiosis, IBR, BHV-4 infection, and neosporosis was 44.88%, 27.34%, 24.41%, 11.72%, and 11.72%, respectively, in cow without any history of abortion. However, the seroprevalences for chlamydiosis, BVD, neosporosis, and BHV-4 infection were 54.17%, 50%, 12.5%, and 4.17%, respectively, in cows with the history of abortion (Table 2). The seroprevalences for BVD and chlamydiosis were significantly different (*P* < 0.05) compared to those for neosporosis and BHV-4 infection in cows that had aborted. None of the cows that had aborted were seropositive for brucellosis, Q fever, or IBR. Coinfection was also detected on 41.67% of cows with the history of abortion (Table 3).

## 4. Discussion

We studied the infectious causes of reproductive diseases in Indonesian cows. The most important infectious causes of reproductive diseases were BVD, chlamydiosis, IBR, neosporosis, and BHV-4 infection. Significant variations of the prevalence of the diseases exist among beef and dairy cows and regions. The seroprevalences for BVD, chlamydiosis, and neosporosis were evenly distributed across all study sites, whereas for IBR and BHV-4 infection tended to be localized at one site. Brucellosis and Q fever were also detected evenly across all study sites, but their overall seroprevalence was below 5%. We recommend including chlamydiosis, neosporosis, and BHV-4 infection in the list of national priority diseases. Prevention and control decisions like vaccination if applicable should be focused in regions with high risk.

### 4.1. Reproductive Diseases in Cows in Indonesia

We revealed five reproductive diseases with high seroprevalences (≥10%), namely, BVD (44.88%), chlamydiosis (27.34%), IBR (24.41%), neosporosis (11.72%), and BHV-4 infection (11.72%). The Ministry of Agriculture of the Republic of Indonesia has been more focused on and has been allocating a large budget for brucellosis monitoring, while reproductive diseases that have high prevalences have been neglected. Chlamydiosis, neosporosis, and BHV-4 infection are not included in the national priority, causing them to receive less attention in reproductive disease monitoring.

The infectious causes of reproductive disease were reported to vary in different countries. For example, three main infectious reproductive disease in Brazil cattle herds were IBR, BVD, and leptospirosis [16]. In addition, neosporosis (21.8–35%) along with IBR (54.4–60.3%) and BVD (30–42.5%) were reported as three main reproductive diseases in Brazil [17]. In Ghana, the most frequent reproductive diseases were IBR (69.9%), BHV-4 infection (21.9%), BVD (15.1%), and Q fever (6.8%) [18]. Different reproductive diseases associated with abortion in dairy cows in Algeria were neosporosis (15%), BHV-4 infection (3.61%), brucellosis (3.06%), Q fever (1.67%), and BVD (1.39%) [11]. Most common reproductive diseases in Argentina were neosporosis (14.67%), brucellosis (6.65%), BHV (2%), and BVD (1.33%) [3].

The reported reproductive diseases with high prevalences (≥10%) in various countries are BVD, IBR, BHV-4 infection, and neosporosis. Our results suggest that BVD, chlamydiosis, and neosporosis have high prevalence and are evenly distributed in at least four locations in this study. The prevalence of neosporosis was significantly (*P* < 0.05) higher in dairy cows than beef cattle. Meanwhile, the prevalence of IBR was significantly (*P* < 0.05) higher in beef cattle than dairy cow and found only in one location, i.e., Pasuruan. On the other hand, the prevalence of BHV-4 infection was significantly (*P* < 0.05) higher in dairy cows and observed only in one location, in Bogor. The exact causes of this discrimination are not yet known.

### 4.2. Reproductive Diseases in Aborted Cows

The most prevalent infectious diseases associated with abortion in Indonesia are chlamydiosis (54.17%), BVD (50%), and neosporosis (12.50%). One study reported IBR, BVD, and neosporosis as the main causes of loss of pregnancy in cattle in Brazil [17]. Other studies reported that neosporosis was proportionally the main cause of loss of pregnancy in cattle with a prevalence of above 15%, while BVD had a prevalence of below 5% [9, 11], and even chlamydiosis was only 0.83% [11]. These results are expected to change the old perspective and belief that the main cause of abortion in Indonesia is *B. abortus*. The evidence we observed was contradictory, that brucellosis was not found in abortive cows as also reported in California, USA [9]. Therefore, chlamydiosis, BVD, and neosporosis require more intensive attention in reproductive disease surveillance in Indonesia.

### 4.3. BVD

BVD is considered a reproductive disease with moderate to high prevalence across the world [19]. Subclinical infections by BVDV in pregnant cows can cause abortion 10–90 days postinfection [13]. BVD is also suspected to be transmitted through the vector *Stomoxys calcitrans* as its genetic material was discovered on flies that fed on persistently infected cattle [20]. This could lead to a wider distribution area. Another disadvantage of BVDV infection is the various congenital defects on the fetus, especially on the central nervous system [19]. Therefore, BVD does not only cause abortions but also congenital defects in various organ systems.

The BVD seroprevalence in Indonesia (44.88%) was the highest. In Indonesia, there are no BVD vaccination programs, so seropositive results in this study and other studies likely originated from virus exposure in the field. Higher BVD prevalence has also been reported in imported cows (63%) that enter Indonesia with no vaccination documents [21]. Its similar prevalence is as found in Belgium and Serbia. The average BVD prevalence in Belgium was reported to be 36.4%, while in Serbia, it was 10.64–74% [22, 23]. Australia, which is an endemic area for BVD, also reports its prevalence of BVD as 13% in South Australia, 65% in Victoria, 35% in Tasmania, and even reaching 92% in the Northern Territory [24].

Based on the results of this study, it was also revealed that BVD seroprevalence in dairy cow and beef cattle in Indonesia was 47.22% and 41.82%, respectively. This result was slightly lower than the results of other studies in Indonesia in dairy cows which ranged from 56.25 to 77% [25, 26]. In contrast, the seroprevalence in beef cattle in this study was higher than that of other studies in Indonesia, ranging from 9.26 to 28% [26, 27]. Overall, there is evidence that BVD prevalence in dairy cow in Indonesia is higher than in beef cattle. The results of this study are contrary to reports in Belgium which stated that BVD prevalence in dairy cow is not different from that of beef cattle, namely, 29.5% and 30.6% [22].

### 4.4. Chlamydiosis

Chlamydiosis associated with abortion is caused by *Chlamydophila abortus* (*C. abortus*) and *Chlamydophila pecorum* (*C. pecorum*) [28–30]. *C. abortus* is widely known to be associated with cases of abortion and mastitis in cattle [29, 31, 32]. The role of *C. pecorum* in abortus is still a matter of debate among researchers. Some researchers claim that *C. pecorum* does not cause abortus but has been known to cause other diseases such as encephalomyelitis, polyarthritis, and keratoconjunctivitis [7, 28, 29]. Others claim that *C. pecorum* can cause abortus, although it is not dominant [12, 30–32].

*C. abortus* has an affinity for placental tissue and thus plays a key role in causing abortion and reproductive disorders, whereas *C. pecorum* is more commonly found in the gastrointestinal tract [29]. This is in line with the study on dairy cows in western Germany which revealed that the sorting order of *Chlamydophila* species causes abortus, i.e., *C. psittaci*, *C. abortus*, and *C. pecorum* with prevalences of 55.80%, 35.80%, and 8.40%, respectively [33].

Based on the results of this study, it was revealed that the cumulative prevalence of chlamydiosis in cows in Indonesia was 27.34%. This result is slightly higher than a previous study (25%) [34]. Similar finding was also reported from Zimbabwe 32.3% [35]. Chlamydiosis seroprevalence in dairy cows in Indonesia is similar to those of Sweden and Italy, which are 28% and 24%, respectively [36, 37]. In contrast, Jordan and Ireland have lower chlamydiosis prevalences in dairy cows at 19.90% and 6.04%, respectively [38, 39]. However, Indonesia's seroprevalence is still lower than that of Taiwan, which reported a chlamydiosis seroprevalence in abortive cows of 71.4% [40].

### 4.5. Neosporosis

Neosporosis seroprevalence in Indonesia has been reported to be 5.80% in Balinese cattle [41]. Another study in 2015 reported that the neosporosis seroprevalences in West Bandung and Bogor were 26.52% and 30%, respectively [42]. Our observation revealed 32% and 27.27% seroprevalences in West Bandung and Bogor, respectively. This evidence indicates that during the period 2015–2020, neosporosis in those two cities tends to be constant.

This study was the first to report a significant difference in the neosporosis seroprevalence between beef and dairy cows in Indonesia, which was 0% and 20.45%, respectively. In Argentina, a significant difference in neosporosis seroprevalence (*P* < 0.01) between beef and dairy cows was reported at 7% and 20.30%, respectively [43]. In Spain, the neosporosis seroprevalence in dairy cows (35.90%) was significantly higher (*P* < 0.01) than in beef cattle (17.90%) [44]. The neosporosis seroprevalence in abortive cows in Belgium was also reported to be significantly different (*P* < 0.05) between beef (14%) and dairy cows (28.60%) [45].

### 4.6. BHV-4 Infection and Brucellosis

This study is the first to report the BHV-4 infection prevalence in cattle in Indonesia. In this study, the BHV-4 infection prevalence in cattle was 11.72%, which was three times higher than that of brucellosis and Q fever. The proportion of BHV-4 infection in aborted cows is 3% (Figure 4) with seroprevalence 4.17% (Table 2). The data are still lower than the USA and Turkey's, which were reported to be 16% and 28.78%, respectively [46, 47]. Turkey reported in detail the BHV-4 infection seroprevalence in repeat-breeder dairy cows and cows with and without reproductive disorders, namely, 53.70% [48], 57.20%, and 44.90%, respectively [49]. The BHV-4 infection data in Indonesia are thought to be lower than the actual value due to the limited sample numbers and sampling area. Therefore, it is advisable to increase the sample numbers and expand the sampling location to obtain more conclusive information.

In general, brucellosis was only detected in dairy cows, especially in Batu (4%), Bogor (13.64%), and West Bandung (8%). Other studies have also reported similar brucellosis seroprevalence in Bogor (17.50%) and Batu (0.77%) [50, 51]. Lower brucellosis seroprevalence in several parts of Indonesia was also reported in Banyuwangi (East Java), which was only 3% and 0% in some areas including Kediri (East Java), Semarang (Central Java), and Boyolali (Central Java) [50, 52, 53]. Exceptionally, brucellosis was reported to be endemic in South Sulawesi province with high seroprevalence (21.90%) [54].

## 5. Conclusion

The dominant reproductive diseases in Indonesian cows are BVD, chlamydiosis, IBR, neosporosis, and BHV-4 infection. Significant variations of the prevalence of the diseases exist among beef and dairy cows and regions. Chlamydiosis, BVD, and neosporosis are common among aborted cows in Indonesia with higher prevalence. Chlamydiosis, neosporosis, and BHV-4 infection are reproductive diseases that are not yet categorized as national priorities in Indonesia. We recommend including these three diseases into the list of strategic infectious diseases as a national priority. Prevention and control decisions like vaccination should be focused in regions with high risk.

## Figures and Tables

**Table 1:** Spatial distribution of bovine reproductive diseases in Indonesian cows^a^.

Regency/city	IBR tested (%)	BHV-4 infection tested (%)	BVD tested (%)	Brucellosis tested (%)	Q fever tested (%)	Chlamydiosis tested (%)	Neosporosis tested (%)	Overall tested (%)
Pasuruan, East Java	29/38 (78.38)	0/38 (0)	21/37 (56.76)	0/38 (0)	1/38 (2.63)	2/38 (5.26)	0/38 (0.00)	53/264 (20.08)
Batu, East Java	0/25 (0)	0/25 (0)	12/25 (48)	1/25 (4)	2/25 (8)	7/25 (28)	1/25 (4)	38/154 (24.68)
Bogor, West Java	2/22 (9.09)	15/22 (68.18)	6/22 (27.27)	3/22 (13.64)	1/22 (4.55)	5/22 (23.81)	6/22 (27.27)	33/175 (18.86)
West Bandung, West Java	0/25 (0)	0/25 (0)	16/25 (64)	2/25 (8)	0/25 (0)	7/25 (28)	8/25 (32)	23/175 (13.14)
Lampung	0/18 (0)	0/18 (0)	2/18 (11.11)	0/18 (0)	0/2 (0)	14/18 (77.78)	0/18 (0)	16/110 (14.56)
Total	31/127 (24.41)	15/128 (11.72)	57/127 (44.88)	6/128 (4.69)	4/112 (3.57)	35/128 (27.34)	15/128 (11.72)	

^a^Based on cows without the history of abortion. IBR, infectious bovine rhinotracheitis; BVD, bovine viral diarrhea; BHV-4 infection, bovine herpesvirus type 4 infection; brucellosis, *Brucella abortus*; Q fever, *Coxiella burnetii*; chlamydiosis, *Chlamydophila abortus*; neosporosis, *Neospora caninum*.

**Table 2:** The seroprevalence of reproductive diseases in cows with and without the history of abortion.

	IBR tested (%)	BHV-4 infection tested (%)	BVD tested (%)	Brucellosis tested (%)	Q fever tested (%)	Chlamydiosis tested (%)	Neosporosis tested (%)	Overall tested (%)
No abortion	31/127 (24.41)	15/128 (11.72)	57/127 (44.88)	6/128 (4.69)	4/112 (3.57)	35/128 (27.34)	15/128 (11.72)	163/878 (18.56)
Aborted cow	0/24 (0)	1/24 (4.17)	12/24 (50)	0/24 (0)	1/24 (4.17)	13/24 (54.17)	3/24 (12.50)	30/168 (17.86)
Total	31/151 (20.53)	16/152 (10.53)	69/151 (45.69)	6/152 (3.95)	5/136 (3.68)	48/152 (31.58)	18/152 (11.84)	

IBR, infectious bovine rhinotracheitis; BVD, bovine viral diarrhea; BHV-4 infection, bovine herpesvirus type 4 infection; Brucellosis, *Brucella abortus*; Q fever, *Coxiella burnetii*; Chlamydiosis, *Chlamydophila abortus*; Neosporosis, *Neospora caninum*.

**Table 3:** The seroprevalence of reproductive diseases in aborted cows.

Coinfection	Tested (%)
BVD + chlamydiosis	6/24 (25)
BVD + BHV-4 infection	1/24 (4.17)
BVD + neosporosis	1/24 (4.17)
Chlamydiosis + Q fever	1/24 (4.17)
BVD + chlamydiosis + neosporosis	1/24 (4.17)
Total	10/24 (41.67)

IBR, infectious bovine rhinotracheitis; BVD: bovine viral diarrhea; BHV-4 infection, bovine herpesvirus type 4 infection; brucellosis, *Brucella abortus*; Q fever, *Coxiella burnetii*; chlamydiosis, *Chlamydophila abortus*; neosporosis, *Neospora caninum*.

## Data Availability

The data used to support the findings of this study are available from the corresponding author upon request.
